# Screening South Asians for type 2 diabetes and prediabetes: (1) comparing oral glucose tolerance and haemoglobin A1c test results and (2) comparing the two sets of metabolic profiles of individuals diagnosed with these two tests

**DOI:** 10.1186/1472-6823-13-8

**Published:** 2013-02-25

**Authors:** Everlina MA Vlaar, Wanda M Admiraal, Wim B Busschers, Frits Holleman, Vera Nierkens, Barend JC Middelkoop, Karien Stronks, Irene GM van Valkengoed

**Affiliations:** 1 Department of Public Health, Academic Medical Centre, University of Amsterdam, Meibergdreef 15, Amsterdam 1105 AZ, The Netherlands; 2 Department of Internal Medicine, Academic Medical Centre, Amsterdam, Netherlands; 3 Department of Public Health, Leiden University Medical Centre, Leiden, The Netherlands; 4 Public Health Service, The Hague, The Netherlands

**Keywords:** Type 2 Diabetes, Prediabetes, South Asian Populations, Screening, HbA1c, OGTT

## Abstract

**Background:**

The glycated haemoglobin A1c (HbA1c) level may be used for screening for type 2 diabetes and prediabetes instead of a more burdensome oral glucose tolerance test (OGTT). However, among the high-risk South Asian population, little is known about the overlap of the methods or about the metabolic profiles of those disconcordantly diagnosed.

**Methods:**

We included 944 South Asians (18–60 years old), whom we screened with the HbA1c level and the OGTT in The Hague, the Netherlands. We calculated the area under the receiver-operator characteristic curve (AUROC) with a 95% confidence interval of HbA1c using the American Diabetes Association classifications, and determined the sensitivity and specificity with 95% confidence intervals at different thresholds. Moreover, we studied differences in metabolic characteristics between those identified by HbA1c and by the OGTT alone.

**Results:**

The overlap between HbA1c and OGTT classifications was partial, both for diabetes and prediabetes. The AUROC of HbA1c for OGTT defined diabetes was 0.86 (0.79–0.93). The sensitivity was 0.46 (0.29–0.63); the specificity 0.98 (0.98–0.99). For prediabetes, the AUROC was 0.73 (0.69–0.77). Each of the 31 individuals with diabetes and 353 with prediabetes identified with the HbA1c level had a high body mass index, large waist circumference, high blood pressure, and low insulin sensitivity, all of which were similar to the values shown by those among the 19 with diabetes or 62 with prediabetes who only met the OGTT criteria, but not the HbA1c criteria.

**Conclusions:**

The HbA1c level identified a partially different group than the OGTT did. However, both those identified with the HbA1c level and those identified with the OGTT alone were at increased metabolic risk.

**Trial registration:**

Dutch Trial Register: NTR1499

## Background

Populations of South Asian origin living in industrialised countries are known to be at high risk of type 2 diabetes and cardiovascular diseases. Thus they form an important target group for active screening and prevention in clinical practice [1-4]. A recent study has shown that such an initiative can potentially provide a substantial benefit in reducing cardiovascular risk [4].

The effectiveness of screening, however, depends in part on the ability of the test method to identify the population at risk. Until recently, the oral glucose tolerance test (OGTT) was recommended for diagnosing diabetes and for detecting individuals at high risk of developing diabetes. In 2010, the American Diabetes Association updated its recommendations to include glycated haemoglobin A1c (HbA1c) at the level of 6.5% or more (≥48 mmol/mol) as a diagnostic option for non-pregnant adults [5]. Because the HbA1c level can be determined with a single blood sample, it has practical advantages and is less burdensome than the OGTT for screening purposes [5]. Further, a number of studies have screened various ethnic populations [6-10]. Most of these studies report that screening based on the HbA1c level may lead to the identification of fewer new cases of diabetes and prediabetes than screening with the OGTT. In this case, there is only a partial overlap [6-10]. Screening with the Hb1Ac level misses some cases that would be detected with the OGTT. Although studies from India suggest that a similar pattern may be expected, such evidence is lacking for South Asians living in industrialised countries [10-12]. This is relevant because the overlap between the HbA1c method and other methods may vary across ethnic groups and across different contexts [6]. Moreover, one recent study shows that the HbA1c levels and OGTT fasting and 2-h glucose levels were higher among South Asians in the UK than among Europeans [13].

A discordance may occur because of measurement variability or because the HbA1c level and the OGTT hallmark different physiological processes [5]. This implies that the metabolic profiles of individuals discordantly diagnosed may differ. Thus, their future health risks may also differ. Indeed, studies among European-origin populations and one study in India have shown that the metabolic profiles differed between those identified with the HbA1c level and those identified with the OGTT only [13-15]. Therefore, among the high-risk South Asian populations living in industrialised countries, it is important to note the characteristics of the metabolic profiles of those who were and those who were not identified with the HbA1c level. The latter group would have been identified with the OGTT.

We evaluated the overlap between classifications and the sensitivity and specificity of HbA1c for diagnosing OGTT-defined diabetes and prediabetes (considering impaired fasting glucose and/or impaired glucose tolerance). The 18 to 60-year-old Hindustani Surinamese participants were screened via family practices in The Hague, the Netherlands. Furthermore, we compared the metabolic profiles of individuals who were HbA1c-identified with the metabolic profile of those whom the OGTT could have identified, but were not HbA1c-identified.

## Methods

### Study design and population

We analysed the data for 944 18 to 60-year-old Hindustani Surinamese participants in a screening program for the DHIAAN study, a culturally targeted, lifestyle-intervention trial. We use the term Hindustani Surinamese to refer to people with South Asian ancestral origin who migrated to the Netherlands via Suriname and their offspring. The Hindustani Surinamese are the descendants of the indentured labourers from North India – Uttar Pradesh, Uttaranchal, and West Bihar – between 1873 and 1917. The two large migration waves, around 1975 and 1980, of Hindustani Surinamese to the Netherlands were caused mainly by the political situation in Suriname [16].

**Figure 1:**
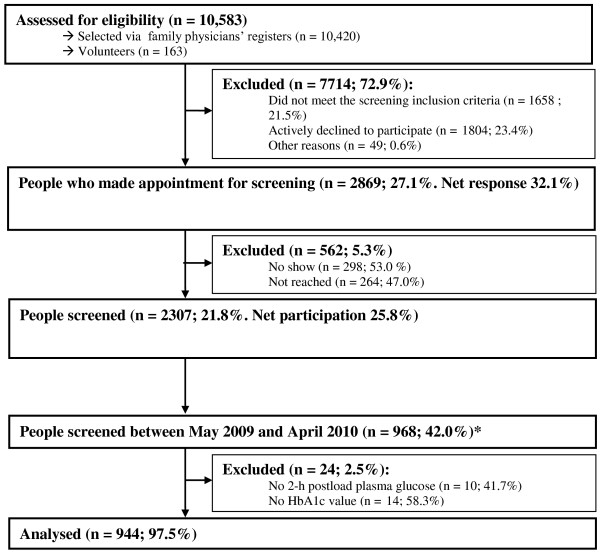
**Flow diagram from eligibility assessment to inclusion in analyses.** Legend: * All participants underwent an oral glucose tolerance test (OGTT) between 18 May 2009 and 19 April 2010. Oral glucose tolerance was no longer tested after April 2010.

The methods of the study have been described elsewhere [17]. In brief, potential participants were selected from 48 family physician lists in The Hague by means of name analysis. All who were not already known to have diabetes received an invitation followed by a written reminder and up to five contact attempts by telephone. Volunteers could also make an appointment for the screening. The participation rate was 21.8% (Figure 1). Because oral glucose tolerance was only tested between 18 May 2009 and 19 April 2010, we only used data for those participating during this period (*n* = 968). Participants who had not completed the OGTT or whose HbA1c measurement was missing were excluded, leaving 944 participants (Figure 1).

The Institutional Review Board of the Academic Medical Centre of the University of Amsterdam approved the study. All participants provided written informed consent.

### Data collection

Participants completed a brief questionnaire about demographics (e.g. country of birth), known risk factors (e.g. family history) and cardiovascular health. Furthermore, a physical examination was carried out. Weight was recorded to the nearest 500 g. Height and waist circumference were measured to the nearest 0.01 m. We calculated the body mass index (BMI; kg/m^2^) from the weight and height. Blood pressure was measured with the participant in the seated position (OmronM5-1, Omron, Hoofddorp, the Netherlands). A maximum of five measurements were taken. We calculated the mean from the first two measurements with less than 5 mmHg difference.

We also asked the participants to donate a fasting blood sample and to undergo an OGTT (the glucose load was 75 g). We analysed the following elements:

1. Fasting plasma glucose. We used the hexokinase method. The samples were collected in BD Vacutainers Fluoride/EDTA Tubes, centrifuged and analysed on a Roche Modular Analytics P (Roche Diagnostics Nederland, Almere, The Netherlands) on the same day.

2. HbA1c. We used high-performance liquid chromatography (DCCT aligned) and analysed the samples on a Tosoh G7 analyser (Tosoh Europe BV, Amsterdam, The Netherlands), and Menarini HA-8160 (A. Menarini Diagnostics Benelux NV, Valkenswaard, The Netherlands) on the day of collection. The variation coefficients were 84 mmol/mol ± 2.0% and 33 mmol/mol ± 2.0%. The Hb variants HbS, HbD, and HbC were automatically reported. HbE and HbJ were verified manually.

3. Insulin. We used the sandwich immunoassay, analysed on a Roche Modular Analytics E170 P (Roche Diagnostics Nederland, Almere, The Netherlands).

Furthermore, a 2-h standard 75-g OGTT was performed. We used the HOMA Calculator (University of Oxford) [18] to determine the insulin sensitivity [in %; homeostasis model assessment (HOMA)-s] and beta cell function (in %; HOMA-b) from fasting plasma glucose and insulin levels (pmol/l).

We used American Diabetes Association recommendations to classify prediabetes and diabetes [5,19]. We defined the criteria for diagnosing diabetes on the basis of the OGTT as a fasting plasma glucose value of 126 mg/dl (7.0 mmol/l) or more and/or a 2-h postload glucose value of 200 mg/dl (11.1 mmol/l). We defined the criteria for diagnosing prediabetes as a fasting plasma glucose value of 100–125 mg/dl (5.6–6.9 mmol/l) and/or a 2-h postload glucose value of 140–199 mg/dl (7.8–11.1 mmol/l) [5,19]. Furthermore, the criterion for diagnosing of diabetes on the basis of the Hb1Ac level was a value of 6.5% (48 mmol/mol) or more, and for prediabetes, an HbA1c level of 5.7%-6.5% (39–48 mmol/mol).

### Statistical analysis

First, we described the population characteristics with medians plus interquartile ranges or percentages. We also calculated the prevalence of diabetes and prediabetes. We determined the overlap between the classifications for diabetes and prediabetes, which we defined as the number of cases identified with both the HbA1c level and the OGTT divided by all cases identified with the HbA1c level.

Second, we calculated the area under the receiver-operator characteristic curve (AUROC) and the 95% confidence intervals for HbA1c to identify OGTT-defined diabetes. We chose the OGTT as a reference on the basis of the local recommendations at the time the study was initiated. We identified the optimal HbA1c threshold for diabetes by interpolating from the AUROC; we selected the point that maximised sensitivity and specificity by inspecting the cross-tabulations of the sensitivity and specificity. We then calculated the sensitivity, specificity, and positive predictive value (PPV) for HbA1c at the recommended and optimal thresholds, with 95% confidence intervals based on bootstrap methods using 2000 samples [20]. After excluding participants with diabetes, we did the same for prediabetes.

Third, we compared those with prediabetes or diabetes according to the recommended thresholds for HbA1c with individuals who only met the recommended OGTT (but not HbA1c) criteria. For this, we used Kruskal-Wallis tests and Mann–Whitney U tests. For reference, we also presented the characteristics of participants whom we could not classify with any of the tests. After excluding all participants with diabetes according to either the HbA1c level or the OGTT, we did the same for prediabetes. We used SPSS 18.0 (Chicago, Illinois) and R2.10.1 (R Foundation for Statistical Computing, 2009) for the statistical analyses.

## Results

The median age of our study population was 43.9 years, 39.4% were male, and 14.5% were born in the Netherlands (Table 1). The prevalences of diabetes based on the OGTT and the HbA1c level were 3.7% (*n* = 35) and 3.3% (*n* = 31), respectively. The prevalence of prediabetes based on the OGTT was 20.2% (*n* = 191), whereas with the HbA1c method, the prevalence of diabetes was 38.8% (*n* = 366). Overall, the overlap for diabetes and prediabetes was partial: 51.6% of the participants with diabetes based on the HbA1c level of 6.5% or more also fulfilled the criteria for diabetes based on plasma glucose. For prediabetes, this was 54.2%. The overlap for HbA1c with impaired fasting glucose (43.0%) was higher than the overlap with impaired glucose tolerance (25.6%).

**Table 1:** Background characteristics and prevalences of type 2 diabetes and prediabetes

	**Total study population*N*** **= 944**
Age in years	43.9 (35.6–51.0)
Number of men (%)	372 (39.4)
Number born in the Netherlands (%)	136 (14.5)
Education: number (%)	
Elementary^a^	125 (13.2)
Intermediate^b^	658 (69.7)
University or equivalent^c^	141 (14.9)
Family with diabetes (%)^d^	690 (73.1)
BMI in kg/m^2^	25.7 (23.1–28.4)
Overweight: 23 ≥ BMI < 27.5 kg/m^2^ (%)	410 (43.4)
Obesity: ≥ 27.5 kg/m^2^ (%)	304 (32.2)
Waist circumference in cm	87.7 (80.7–95.3)
Number with hypertension (%)^e^	295 (31.3)
Systolic blood pressure in mmHg	125 (114–138)
Diastolic blood pressure in mmHg	80 (73–88)
Plasma glucose in mmol/l	
Fasting	4.9 (4.6–5.4)
2 h	5.3 (4.4–6.6)
HbA1c	
in mmol/mol	38 (34–40)
in %	5.6 (5.3–5.8)
Insulin in pmol/l	73.6 (50.0–104.9)
HOMA–S in %^f^	72.1 (51.6–105.3)
HOMA–B in %^f^	118.7 (92.7–152.3)
OGTT-defined type 2 diabetes (%)^g^	35 (3.7)
OGTT-defined prediabetes (%)^h^	191 (20.2)
HbA1c-defined type 2 diabetes (%)^i^	31 (3.3)
HbA1c-defined prediabetes (%)^j^	366 (38.8)

Data are presented as medians (interquartile range) or *n* (percentages).

^a^Primary education or less.

^b^Low vocational training, lower secondary education, intermediate vocational training and higher secondary education.

^c^Higher vocational training or university.

^d^First and second grade.

^e^ Systolic blood pressure ≥ 140 mmHg and/or of distolic blood pressure ≥ 90 mmHg or if hypertension medication;

^f^HOMA-S and HOMA-b were determined from fasting plasma glucose and insulin levels (pmol/l) with the HOMA Calculator (University of Oxford) [16].

^g^OGTT diagnosis of type 2 diabetes: fasting plasma glucose ≥ 126 mg/dl (7.0 mmol/l) and/or 2-h plasma glucose ≥ 200 mg/dl (11.1 mmol/l).

^h^OGTT diagnosis of prediabetes: fasting glucose value of 100–125 mg/dl (5.6 – 6.9 mmol/l) and/or a 2-h postload glucose value of 140–199 mg/dl (7.8–11.1 mmol/l).

^i^HbA1c diagnosis of type 2 diabetes: HbA1c ≥ 6.5% (≥ 48 mmol/mol).

^j^HbA1c diagnosis of prediabetes: 5.7% ≤ HbA1c < 6.5% (39 ≤ HbA1c < 48 mmol/mol).

*BMI*, body mass index; *HbA1c*, glycated haemoglobin *A1c*; HOMA, homeostasis model assessments; *OGTT*, oral glucose tolerance test.

The AUROC for HbA1c as a predictor of OGTT-defined diabetes was 0.86 (0.79–0.93). At the recommended threshold (≥ 6.5%), the sensitivity of HbA1c as a predictor of OGTT-defined diabetes was 0.46 (0.29–0.63); the specificity, 0.98 (0.98–0.99); and the PPV 0.52 (0.35–0.69). The optimal HbA1c threshold for diabetes was 6.3% (45 mmol/mol). At this threshold we found a sensitivity of 0.63 (0.49–0.77), a specificity of 0.96 (0.95–0.97), and a PPV of 0.37 (0.25–0.49). The AUROC for prediabetes was 0.73 (0.69–0.77). At the recommended HbA1c threshold as a predictor of OGTT-defined prediabetes, the sensitivity, specificity, and PPV were 0.66 (0.59–0.73), 0.68 (0.64–0.71), and 0.35 (0.30–0.40), respectively. The optimal range for HbA1c for predicting prediabetes was 5.8–6.3% (40–45 mmol/mol), with a sensitivity of 0.58 (0.51–0.65), a specificity of 0.78 (0.75–0.81), and a PPV of 0.41 (0.35–0.47).

As expected, individuals not diagnosed with diabetes or prediabetes had better metabolic profiles with lower blood pressures, lower BMIs, smaller waist circumferences, and higher median HOMA-s and HOMA-b than those diagnosed as having diabetes or prediabetes (*p* < 0.05 for all characteristics; Table 2). We did not find statistically significant differences between those with diabetes based on the HbA1c level and those who would have been identified solely with the OGTT, apart from differences in fasting plasma glucose and postload plasma glucose (Table 2). Those diagnosed with prediabetes with the HbA1c level did not differ from those meeting only the oral glucose tolerance criteria, apart from differences in HbA1c, fasting plasma glucose, and postload plasma glucose. Nevertheless, those diagnosed with both the HbA1c level and the OGTT had worse metabolic profiles than those identified with the HbA1c level alone (Additional file 1).

**Table 2:** Differences in characteristics according to diagnosis of type 2 diabetes and prediabetes based on the oral glucose tolerance test and the haemoglobin A1c level

**Type 2 diabetes**	**Nondiabetic**	**HbA1c**	**OGTT only**	**p-value for HbA1c vs. OGTT only**
	**(OGGT-/Hba1c-)*n*** **= 894**	**(HbA1c+)*n =*** **31**	**(OGTT+/HbA1c-)*n*** **= 19**	
FPG in mmol/l ^a^	4.9 (4.6–5.3)	6.7 (5.5–7.4)	5.7 (5.3–6.5)	<0.05
2 hour PG in mmol/l ^a^	5.2 (4.4–6.3)	9.2 (5.9–12.8)	12.0 (11.4–12.4)	<0.05
HbA1c in %^a^	5.6 (5.3–5.8)	6.8 (6.6–7.1)	5.9 (5.6–6.3)	0.06
HbA1c in mmol/mol ^a^	38 (34–40)	51 (49–54)	41 (38–45)	0.06
Age in years	43.4 (35.0–50.3)	46.0 (38.3–56.3)	48.7 (43.1–52.3)	0.43
Male	350 (39.1)	12 (38.7)	10 (52.6)	0.72
BMI in kg/m2	25.5 (23.0–28.2)	28.6 (25.4–29.9)	27.9 (24.8–30.0)	0.68
Waist circumference in cm	87.2 (80.3–95.0)	97.2 (90.0–102.3)	92.0 (88.3–100.0)	0.35
Systolic blood pressure in mmHg	124 (114–136)	134 (117–144)	125 (118–143)	0.67
Diastolic blood pressure in mmHg	80 (73–88)	86 (82–96)	82 (74–94)	0.20
Insulin in pmol/l	70.8 (47.9–100.7)	120.1 (75.0–190.0)	128.5 (90.6–195.0)	0.63
HOMA-s in %^b^	75.4 (53.5–108.7)	44.5 (28.2–67.9)	40.7 (27.2–59.0)	0.67
HOMA-b in %^b^	119.3 (93.2–152.0)	112.5 (70.9–138.5)	122.9 (91.5–180.3)	0.17
**Prediabetes**^**c**^	**Non-pre-diabetic**	**HbA1c**	**OGTT only**	**p-value**
	**(OGTT-/HbA1c-),*n*** **= 479**	**HbA1c +)*n*** **= 353**	**(OGTT+/HbA1c-)*n*** **= 62**	
FPG in mmol/l*^a^	4.8 (4.5–5.0)	5.2 (4.9–5.6)	5.6 (4.9–5.8)	<0.05
2 hour PG in mmol/l ^a^	4.9 (4.2–5.8)	5.9 (4.8–7.2)	7.0 (5.4–8.2)	<0.05
HbA1c in %^a^	5.4 (5.2–5.5)	5.9 (5.7–6.0)	5.4 (5.2–5.6)	<0.05
HbA1c in mmol/mol ^a^	35 (33–37)	41 (39–42)	36 (33–38)	<0.05
Age in years	40.1 (31.1–47.1)	48.1 (41.3–53.4)	46.0 (39.4–52.1)	0.19
Male in %	163 (34.0)	154 (43.6)	32 (51.6)	0.06
BMI in kg/m2	24.7 (22.2–27.3)	26.5 (24.1–29.6)	26.5 (23.4–29.3)	0.74
Waist circumference in cm	83.7 (77.2–91.0)	90.5 (84.9–99.1)	92.2 (84.7–99.6)	0.66
Systolic blood pressure in mmHg	120 (110–131)	128 (118–142)	129 (117–137)	0.61
Diastolic blood pressure in mmHg	77 (71–84)	82 (76–90)	81 (75–88)	0.26
Insulin in pmol/l	67.4 (45.1–95.1)	79.1 (54.2–116)	79.9 (62.5–116.5)	0.54
HOMA-s in %^b^	79.2 (57.9–117.5)	67.2 (46.7–98.4)	65.6 (45.4–83.1)	0.47
HOMA-b in %^b^	122.3 (96.2–156.0)	113 (81.1–151.2)	112.7 (85.5–138.3)	0.43

Data are presented as medians (interquartile range) or *n* (percentages).

^a^Variables on which the classification of prediabetes and type 2 diabetes was based.

^b^HOMA-s and HOMA-b were determined from FPG and insulin levels (pmol/l) with the HOMA Calculator (University of Oxford) [16].

^c^All participants with type 2 diabetes according to either HbA1c or OGTT were excluded from the analyses for prediabetes.

*BMI*, body mass index; *FPG*, fasting plasma glucose; *HbA1c*, glycated haemoglobin A1c; *OGTT*, oral glucose tolerance test; *PG*, plasma glucose.

## Discussion

We found that we identified fewer new cases of diabetes when we used the HbA1c method, but more new cases of prediabetes than we did when we used the OGTT in our population of South Asian origin. Moreover, the overlap of the classifications based on the HbA1c level and the OGTT was only partial, and the sensitivity and specificity (prediabetes only) of HbA1c for identifying OGTT-defined diabetes and prediabetes were low. Regardless of the partial overlap, we found that all those identified had poor metabolic profiles. The metabolic profiles of those identified with the HbA1c level and those identified with the OGTT alone did not differ. This was the case for both diabetes and prediabetes.

Although the AUROC in our study was high, the low sensitivity of HbA1c for OGTT-defined diabetes and prediabetes was in line with previous studies in various ethnic groups [8,10,11,21-23]. For instance, a recent cost analysis in a South Asian population aged 40–75 years in the UK reports low sensitivity of HbA1c (at >6.5%) for diabetes [23]. Although many studies have reported a relatively high specificity, a study in India – similarly to our study – found low specificity of HbA1c, particularly for prediabetes [8,10,11,21-23]. The lower optimal HbA1c threshold for prediabetes in our population was in line with the threshold that the Indian study recommended [10]. Moreover, the lower optimal HbA1c threshold corresponds to the American Diabetes Association’s current recommendations [19]. Yet, the continued low sensitivity and specificity for this lower threshold seems to support the World Health Organisation’s recommendation not to use HbA1c to determine prediabetes [24].

Data characterising participants discordantly categorised with the HbA1c level and the OGTT in South Asian populations in industrialised countries is lacking. One study in India suggests that, as they did in our study, the use of A1c criteria would identify a different set of individuals with milder glucose intolerance [12]. This Indian study has also found lower serum triglyceride levels among those diagnosed with the HbA1c level [12]. Previous studies in other populations have been inconsistent. Our results appear to be in line with some studies reporting that those diagnosed with the HbA1c level had as unfavourable metabolic profiles as those identified with the OGTT [15,25]. However, in contrast to our results, Boronat et al. report that individuals newly diagnosed with diabetes who meet the HbA1c criteria for diabetes have higher BMIs, higher HOMA insulin resistance, and lower HDL (high density lipoprotein) cholesterol than individuals fulfilling the OGTT criteria only [14]. The difference between the Indian study and ours is likely related to differences in the overlap of the measurements associated with the ethnic background of the study population or the setting in which the study took place.

Notably, we found that all individuals identified should be considered at risk of adverse outcomes, regardless of the criterion used [26-28]. For instance, those identified in our study with the HbA1c level or with the OGTT alone all had relatively high BMIs and waist circumferences. This is particularly important for those with prediabetes. Previous studies have shown that South Asians develop complications of obesity, such as diabetes, at lower thresholds for BMI and waist circumferences than individuals of European origin [29,30]. To identify all individuals at risk of adverse outcomes, a strategy that combines more than one diagnostic method should perhaps be considered for this population instead of the strategy of choosing between the HbA1c level and the OGTT. However, this should also depend on the association of either criterion with the occurrence of complications. A combined testing strategy may not be feasible or acceptable in the local context, e.g. because of budget restrictions.

### Limitations

Our study has a few limitations that merit discussion. The first one is the relatively low participation rate, even though it is higher than the participation rate in two recent studies among populations of South Asian origin selected from general practices in the UK [4,31]. This may be related to the recruitment strategy in our study, which was more intensive than the strategies of the UK studies. Our results may have been affected if the participation was selective. We did find evidence for a relatively lower response rate among men and younger participants. However, this is not likely to have greatly influenced our results: previous studies have not consistently found differences in the relationship between HbA1c- and OGTT-diagnosed diabetes when the characteristics sex and age have been correlated [32,33].

The second limitation of our study is the relatively low prevalence of newly diagnosed diabetes, which may have influenced the power of the analyses. Therefore, a lack of significant differences between individuals with OGTT- and HbA1c-diagnosed diabetes may reflect a true lack of difference, but may also be the result of lack of power to demonstrate actual differences. In addition, the small number of individuals diagnosed implies that no adjustment could be made for relevant parameters, such as sex and age, in the comparisons of characteristics. The low prevalence may partly be a result of the intensified case finding recommended for this population in the guideline of the Dutch College of General Practitioners [34]. Given that this guideline is based on fasting plasma glucose, the recommended case finding might have also affected the reported overlap of the classifications based on the HbA1c level and the OGTT.

Finally, we might not have measured all the relevant metabolic parameters. For instance, other studies have found differences in HDL [14,15]. In contrast to other studies, we could not take lipid profiles into account because they were not measured during the initial screening.

## Conclusions

Classifications of diabetes and prediabetes with the HbA1c level and the OGTT only partially overlapped among the 18 to 60-year-old South Asian population in our study. Importantly, both those identified with the HbA1c level and those identified with the OGTT alone had adverse metabolic profiles. These two groups form an important target group for preventive interventions to reduce their future health risks. This implies that it may be worthwhile to consider a strategy in which these diagnostic methods are combined for South Asian populations. Therefore, if it proves feasible in the local context, we recommend that future studies evaluate the efficiency of different diagnostic strategies for the uptake, the number of identifiable cases, and potential gains in terms of averted health risks.

## Abbreviations

AUROC: Area under the receiver operating characteristic curve; BMI: Body mass index; FPG: Fasting plasma glucose; HbA1c: Glycated haemoglobin A1c; HDL: High density lipoprotein; HOMA: Homeostasis model assessment; OGTT: Oral glucose tolerance test; PG: Plasma glucose; PPV: Positive predictive value.

## Competing interests

The authors declare that they have no competing interests.

## Authors’ contributions

EV and WA participated in the analyses, contributed to the interpretation, and drafted the manuscript. WB analysed the data and contributed to the interpretation. BM and VN were consulted for the design and interpretation, and they reviewed the manuscript. IV and KS contributed to the design of the study, helped interpret the data, and reviewed and edited the manuscript. All authors read and approved the final manuscript.

## Pre-publication history

The pre-publication history for this paper can be accessed here:

http://www.biomedcentral.com/1472-6823/13/8/prepub

## Supplementary Material

Additional file 1 **Comparison single versus both criteria-revised.** Title of data: Differences in characteristics according to diagnosis of type 2 diabetes and prediabetes based on OGTT and HbA1c. Description of data: In this file we report on the metabolic characteristics of those diagnosed with the HbA1c level and the OGTT versus those diagnosed with the HbA1c level alone or with the OGTT alone. Click here for file
