# Supplementary material for: Screening South Asians for type 2 diabetes and prediabetes: (1) comparing oral glucose tolerance and haemoglobin A1c test results and (2) comparing the two sets of metabolic profiles of individuals diagnosed with these two tests
Source: BMC Endocr Disord. 2013 Feb 25;13:8. doi: 10.1186/1472-6823-13-8 (PMC3700889; doi:10.1186/1472-6823-13-8)
Supplement: Additional file 1 — Comparison single versus both criteria-revised. Title of data: Differences in characteristics according to diagnosis of type 2 diabetes and prediabetes based on OGTT and HbA1c. Description of data: In this file we report on the metabolic characteristics of those diagnosed with the HbA1c level and the OGTT versus those diagnosed with the HbA1c level alone or with the OGTT alone. [file 1472-6823-13-8-S1.doc]

**Table 1.** Differences in characteristics according to diagnosis of type 2 diabetes and prediabetes based on oral glucose tolerance test and the glycated haemoglobin A1c level

| **Type 2 diabetes** | **Both Criteria**  (OGGT+/Hba1c+)  *n* = 16 | **HbA1c only**  (HbA1c+/OGTT-),  *n* = 15 | **OGTT only**  (OGTT+/HbA1c-),  *n* = 19 | **p-value**  **HbA1c only vs. both criteria** | **p-value**  **OGGT only vs. both criteria** |
| --- | --- | --- | --- | --- | --- |
| FPG in mmol/l ^a^ | 7.4 (7.0–7.6) | 5.3 (5.5–5.9) | 5.7 (5.3–6.5) | <0.05 | <0.05 |
| 2 hour PG in mmol/l ^a^ | 13.0 (11.7–16.3) | 6.1 (5.2–8.0) | 12.0 (11.4–12.4) | <0.05 | 0.08 |
| HbA1c in %^a^ | 6.9 (6.8–7.3) | 6.7 (6.5–6.8) | 5.9 (5.6–6.3) | <0.05 | <0.05 |
| HbA1c in mmol/mol ^a^ | 52 (51–56) | 50 (48–51) | 41 (38–45) | <0.05 | <0.05 |
| Age in years | 49.0 (40.1–50.9) | 50.9 (44.9–54.7) | 46.0 (38.3–56.3) | 0.21 | 0.72 |
| Male | 9 (56.3) | 3 (20.0) | 10 (52.6) | <0.05 | 0.56 |
| BMI in kg/m^2^ | 28.0 (23.7–29.2) | 29.4 (26.6–30.4) | 27.9 (24.8–30.0) | 0.14 | 0.66 |
| Waist circumference in cm | 99.7 (87.4–103.7) | 97.0 (94.2–102) | 92.0 (88.3–100.0) | 0.90 | 0.29 |
| Systolic blood pressure in mmHg | 143 (117–150) | 126 (117–134) | 125 (118–143) | <0.05 | 0.22 |
| Diastolic blood pressure in mmHg | 93 (84–98) | 84 (82–88) | 82 (74–94) | 0.07 | 0.08 |
| Insulin in pmol/L | 156.3 (73.4–223.1) | 117.0 (76.4–166.0) | 128.5 (90.6–195.0) | 0.23 | 0.77 |
| HOMA-s in %^b^ | 32.5 (22.9–68.0) | 50.0 (32.9–67.9) | 40.7 (27.2–59.0) | 0.14 | 0.57 |
| HOMA-b in %^b^ | 95.7 (54.6–120.5) | 120.2 (85.5–147.1) | 122.9 (91.5–180.3) | 0.08 | <0.05 |
| **Prediabetes^c^** | **Both Criteria**  (OGTT-/ HbA1c-)  *n* = 123 | **HbA1c only**  (HbA1c +, OGTT-)  *n* = 230 | **OGTT only**  (OGTT+/HbA1c-)  *n* = 62 | **p-value**  **HbA1c only vs both criteria** | **p-value**  **OGGT only vs both criteria** |
| FPG in mmol/l*^a^ | 5.7 (5.4–5.9) | 5.0 (4.7–5.2) | 5.6 (4.9–5.8) | <0.05 | <0.05 |
| 2 hour PG in mmol/l ^a^ | 7.7 (5.8–8.5) | 5.4 (4.5–6.3) | 7.0 (5.4–8.2) | <0.05 | 0.09 |
| HbA1c in %^a^ | 6.0 (5.8–6.1) | 5.8 (5.7–5.9) | 5.4 (5.2–5.6) | <0.05 | <0.05 |
| HbA1c in mmol/mol ^a^ | 42 (40–42) | 40 (39–41) | 36 (33–38) | <0.05 | <0.05 |
| Age in years | 48.9 (41.7–54.1) | 47.5 (40.7–53.1) | 46.0 (39.4–52.1) | 0.15 | 0.06 |
| Male in % | 66 (53.7) | 88 (38.3) | 32 (51.6) | <0.05 | 0.46 |
| BMI in kg/m^2^ | 27.7 (24.7–29.7) | 25.9 (23.8–29.0) | 26.5 (23.4–29.3) | <0.05 | 0.09 |
| Waist circumference in cm | 95.0 (87.1–102.6) | 89.2 (83.2–96.3) | 92.2 (84.7–99.6) | 0.05 | 0.10 |
| Systolic blood pressure in mmHg | 130 (121–146) | 128 (116–138) | 129 (117–137) | 0.05 | 0.15 |
| Diastolic blood pressure in mmHg | 86 (76–91) | 82 (116–138) | 81 (75–88) | 0.05 | 0.06 |
| Insulin in pmol/l | 91.0 (62.3–144.3) | 73.6 (51.4–106.6) | 79.9 (62.5–116.5) | <0.05 | 0.17 |
| HOMA-s in %^b^ | 56.9 (37.0–83.0) | 74.0 (51.2–104.3) | 65.6 (45.4–83.1) | <0.05 | 0.13 |
| HOMA-b in%^b^ | 107.2 (80.6–146.7) | 116.6 (96.1–152.2) | 112.7 (85.5–138.3) | <0.05 | 0.68 |

Data are presented as medians with interquartile ranges or *n* in percentages

^a^Variables on which the classification of prediabetes and type 2 diabetes was based

^b^ HOMA-s and HOMA-b were determined from FPG and insulin levels (pmol/l) with the HOMA Calculator (University of Oxford) [16]

^c^All participants with type 2 diabetes according to either HbA1c or OGTT were excluded from the analyses for prediabetes

BMI, body mass index; FPG, fasting plasma glucose; HbA1c, glycated haemoglobin A1c; OGTT, oral glucose tolerance test; PG, plasma glucose
